# Supplementary material for: The Association between Iron Deficiency and Renal Outcomes Is Modified by Sex and Anemia in Patients with Chronic Kidney Disease Stage 1–4
Source: J Pers Med. 2023 Mar 14;13(3):521. doi: 10.3390/jpm13030521 (PMC10057396; doi:10.3390/jpm13030521)
Supplement: Supplementary file 1 [file jpm-13-00521-s001.zip › jpm-2257565-supplementary.pdf]

Table S1. Multivariate linear regression (fully-adjusted model) for serum iron per 1 SD change

Variables	β coefficient	95% CI	*P* value
Age (per 1 SD)	-0.001	-0.003 to 0.003	0.964
Female (vs male)	-0.238	-0.324 to -0.152	<0.001
eGFR (per 1 SD)	0.001	-0.001 to 0.003	0.473
Log UPCR (per 1 SD)	0.064	-0.013 to 0.140	0.104
DM	-0.282	-0.365 to -0.198	<0.001
CVD	-0.088	-0.194 to 0.018	0.104
Cancer	0.006	-0.138 to 0.151	0.931
Smoker	0.001	-0.111 to 0.113	0.989
Severe liver disease	0.106	-0.093 to 0.306	0.296
Hypertension	0.006	-0.074 to 0.086	0.879
Hb (per 1 SD)	0.123	0.101 to 0.146	<0.001
Log cholesterol	-0.235	-0.588 to 0.117	0.191
Mean BP (per 1 SD)	-0.005	-0.008 to -0.003	<0.001
Body mass index (per 1 SD)	-0.009	-0.019 to 0.000	0.061
Albumin (per 1 SD)	0.150	0.051 to 0.249	0.003
Log CRP (per 1 SD)	0.001	-0.044 to 0.046	0.968
Phosphorus (per 1 SD)	0.075	0.025 to 0.124	0.003
Malnutrition-inflammation*			
Male	-0.052	-0.078 to -0.025	<0.001
Female	-0.003	-0.032 to 0.025	0.807

Abbreviations: SD, standard deviation; CI, confidence interval; eGFR, estimated glomerular filtration rate; Log, log-transformation; UPCR, urine protein to creatinine ratio; DM, diabetes mellitus; CVD, cardiovascular disease; Hb, hemoglobin; BP, blood pressure; CRP, C-reactive protein. * Presence of malnutrition –inflammation was indicated by malnutrition-inflammation score. P for interaction <0.05 stratifeid by gender.
